# Two cases of lung neuroendocrine carcinoma with carcinoid morphology

**DOI:** 10.1186/s13000-019-0886-1

**Published:** 2019-09-12

**Authors:** Kenji Inafuku, Tomoyuki Yokose, Hiroyuki Ito, Daisuke Eriguchi, Joji Samejima, Takuya Nagashima, Haruhiko Nakayama, Masaki Suzuki, Kouzo Yamada, Munetaka Masuda

**Affiliations:** 1 Department of Thoracic Surgery, Kanagawa Cancer Center, 2-3-2 Nakao, Asahi-ku, Yokohama City, Kanagawa 241-8515 Japan; 2 Department of Pathology, Kanagawa Cancer Center, 2-3-2 Nakao, Asahi-ku, Yokohama City, Kanagawa 241-8515 Japan; 3 Department of Thoracic Oncology, Kanagawa Cancer Center, 2-3-2 Nakao, Asahi-ku, Yokohama City, Kanagawa 241-8515 Japan; 4 Department of Surgery, Yokohama City University, 3-9 Fukuura, Kanazawa-ku, Yokohama City, Kanagawa 236-0004 Japan

**Keywords:** Carcinoid morphology, Lung tumor, Neuroendocrine carcinoma, Neuroendocrine tumor grade 3, Proliferative activity

## Abstract

**Background:**

The category of grade 3 neuroendocrine tumor (NET G3) was newly introduced in the 2017 World Health Organization (WHO 2017) classification of neuroendocrine neoplasms of the pancreas. Pancreatic NET G3 shows a carcinoid-like morphology with high proliferative activity and the prognosis is intermediate between NET G2 and neuroendocrine carcinoma. There is no category corresponding to NET G3 in the current WHO 2015 classification of lung tumors. Herein, we report two cases of lung neuroendocrine carcinoma with carcinoid morphology that correspond to NET G3.

**Case presentation:**

Case 1: An abnormal chest shadow was detected in a 78-year-old female never-smoker during a routine medical examination. She was asymptomatic. The radiological assessment revealed a mass in the peripheral S4 segment of the right lung. She underwent right middle lobectomy for the mass preoperatively diagnosed as non-small cell lung carcinoma. Postoperative histological examination revealed a neuroendocrine tumor with carcinoid morphology and a mitotic count of 15/2 mm^2^. Case 2: An abnormal chest shadow was detected in a 74-year-old female never-smoker undergoing follow-up for another disease. She was asymptomatic. The radiological assessment revealed a mass in the peripheral S3 segment of the right lung. She underwent right upper lobectomy for the mass suspected to be lung carcinoma. Postoperative histological examination revealed a neuroendocrine tumor with carcinoid morphology with mitotic count of 13/2 mm^2^. Both of these tumors showed carcinoid morphology but with a mitotic count exceeding 10/2 mm^2^; thus, we diagnosed them as small cell lung carcinomas according to the current WHO 2015 classification.

**Conclusions:**

Our tumors occurred in female never-smokers and their histology showed carcinoid morphology without extensive necrosis. Moreover, proliferative abilities of them were extremely low compared to small cell lung carcinoma. The clinical and pathological features of our tumors appeared to be different from those of small cell lung carcinoma. Although there is no category corresponding to NET G3 in the current classification of lung tumors, we consider that our tumors may correspond to NET G3 and identification of this subset is relevant for therapeutic management.

## Background

The 2017 World Health Organization (WHO 2017) classification of tumors separates pancreatic neuroendocrine neoplasms (NENs) into two broad categories—NEN with carcinoid-like morphology and poorly differentiated NEN—and has incorporated a new subclassification to the high-grade NEN with carcinoid-like morphology category, grade 3 neuroendocrine tumor (NET G3). NET G3 shows a carcinoid-like morphology with high proliferative activity and the prognosis is intermediate between NET G2 and neuroendocrine carcinoma (NEC) [1]. This new category algorithm aims to improve outcomes and help better therapeutic strategies for patients.

In the WHO 2015 classification of lung tumors, NETs are classified as low-grade typical carcinoid (TC), intermediate-grade atypical carcinoid (AC), high-grade large cell neuroendocrine carcinoma (LCNEC), and high-grade small cell lung carcinoma (SCLC) with no NET G3 category [2, 3]. Here we report two cases of lung NEC with carcinoid morphology having a mitotic count of > 10/2 mm^2^ that better correspond to NET G3.

## Case presentation

### Case 1

**Fig. 1:**
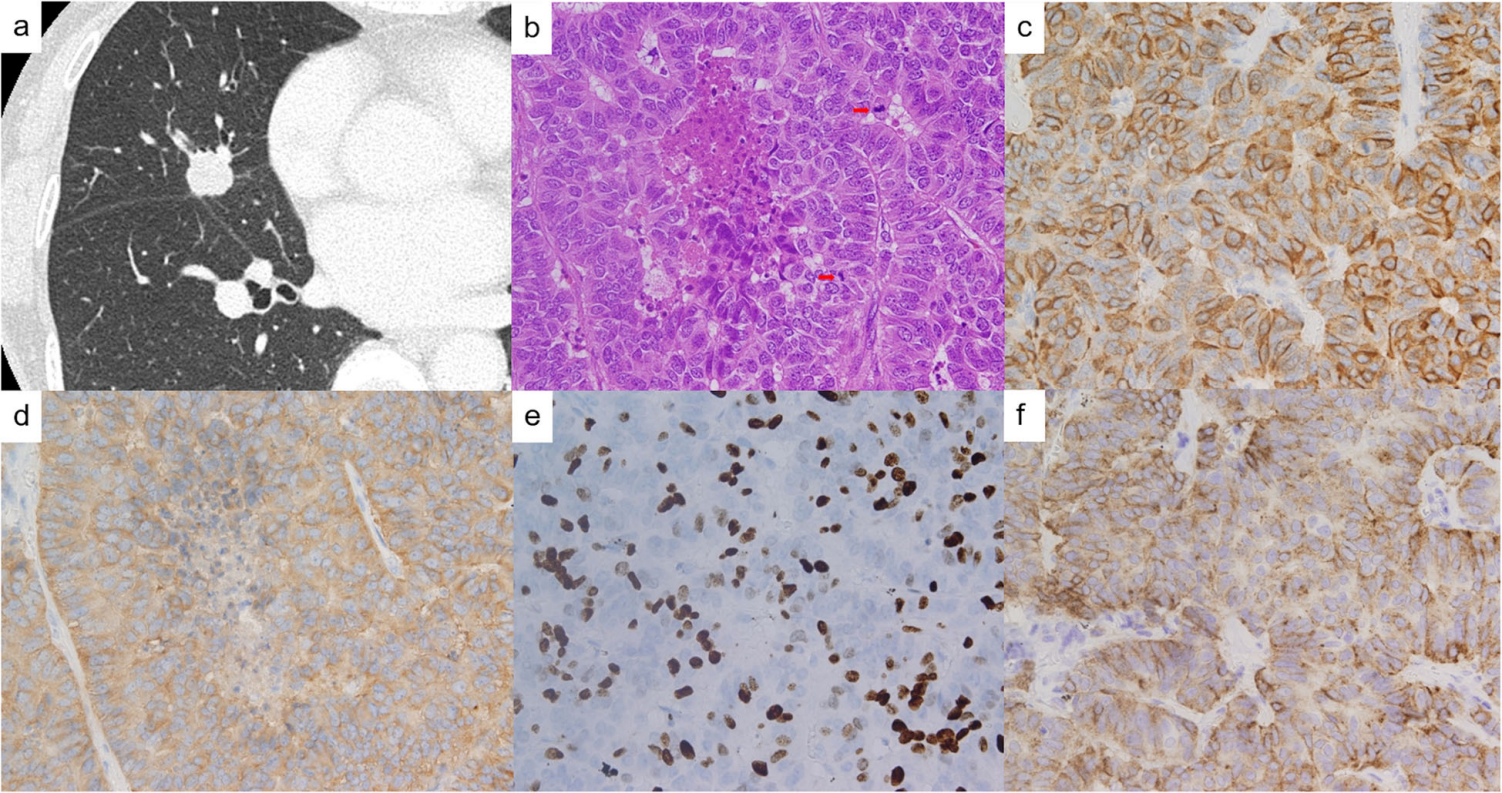
Radiological image and histopathological findings in case 1. **a** CT showed a 1.6-cm mass in the peripheral zone of right S4. **b** Solid growth pattern was seen with central small necrosis. Two mitotic figures (red arrows) were observed (hematoxylin and eosin stain, × 40). **c** Tumor cells found positive for chromogranin A. **d** Tumor cells found positive for synaptophysin. **e** 40% of tumor cells found positive for Ki67. **f** SSTR-2 staining showed presence of a membranous pattern of staining in less than 50% of tumor cells (score 2). +−

An abnormal chest shadow was detected in a 78-year-old asymptomatic female during a routine medical examination. She was a never-smoker and her past history included hypertension. The physical examination showed no abnormalities. Chest computed tomography (CT) imaging showed a 1.6-cm mass in the peripheral S4 segment of the right lung (Fig. 1a). Her pro-gastrin-releasing peptide (proGRP) level had elevated to 324 ng/mL. Preoperative 18F-fluorodeoxyglucose-positron emission tomography (FDG-PET) showed accumulation of FDG in the tumor (the maximum standardized uptake value (SUVmax) of 6.0). A transbronchial lung biopsy (TBLB) showed non-small cell lung carcinoma, which was identified as stage IA2 (cT1bN0M0). A right middle lobectomy and lymph-node dissection were performed.

Macroscopically, the tumor was a well-circumscribed white solid mass, measuring 1.7 cm in diameter with pleural indentation. Histologically, the round-to-spindle tumor cells with a high nucleus-to-cytoplasm ratio and fine nuclear chromatin had neuroendocrine morphology such as pseudoglandular, trabecular, ribbon-like and solid patterns (Fig. 1b). Central small necrosis and peripheral rosette-like structures presented in the solid nest. Lymphovascular and pleural invasion were identified and the mitotic count was 15/2 mm^2^.

Immunohistochemically, the tumor cells were positive for neuroendocrine markers (chromogranin A, synaptophysin, CD56) and thyroid transcription factor-1 (TTF-1) (Fig. 1c, d). The Ki-67 index for the evaluation of proliferative ability was 40% (counting at least 500 tumor cells in hot spots using digital image analysis software) (Fig. 1e). Immunostaining of somatostatin receptor (SSTR)-2, which is overexpressed in well-differentiated NENs, showed the presence of a membranous pattern of staining in less than 50% of tumor cells (score 2) (Fig. 1f) [4]. We diagnosed this tumor as SCLC according to WHO 2015 classification of lung tumors. The pathologic stage was IB (T2aN0M0).

She did not receive adjuvant chemotherapy. She was followed up by regular evaluations such as a physical examination, a blood examination, and CT. As of 22 months after surgery, she remains in good health without relapse of lung cancer.

### Case 2

**Fig. 2:**
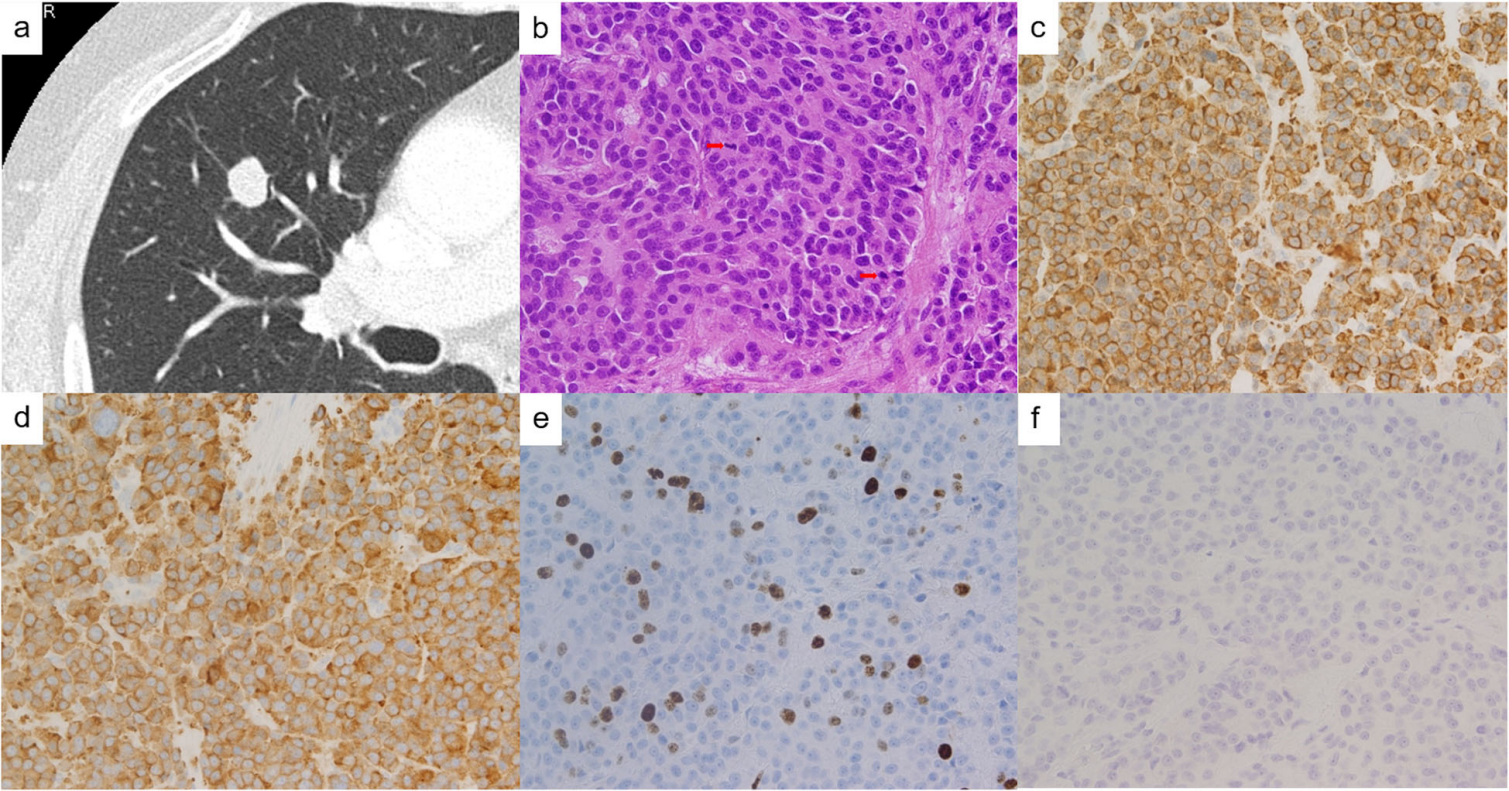
Radiological image and histopathological findings in case 2. **a** CT showed a 1.4 cm mass in the peripheral zone of right S3. **b** Solid proliferation of round-to-ovoid tumor cells were seen and two mitotic figures (red arrows) can be seen in this hematoxylin and eosin staining micrograph (× 40). **c** Tumor cells found positive for chromogranin A. **d** Tumor cells found positive for synaptophysin. **e** 27% of tumor cells were positive for Ki67. **f** Tumor cells found absent of SSTR-2 immunoreactivity (score 0)

An abnormal chest shadow was detected in a 74-year-old asymptomatic female never-smoker undergoing follow-up for another disease. Her past history included hypertension and gastroduodenal ulcer. The physical examination showed no abnormalities. CT imaging showed a 1.4-cm mass in the peripheral S3 segment of the right lung (Fig. 2a). Her blood examination, including tumor markers, were within normal limits. FDG-PET scan showed accumulation of FDG in the tumor (SUVmax of 2.83). Although the TBLB showed no malignancy, the mass was suspected to be lung cancer in clinical T1bN0M0 stage IA2; a right upper lobectomy and lymph-node dissection were performed.

Macroscopically, the tumor was a well-circumscribed white solid mass, measuring 1.3 cm in diameter. Histological findings showed the solid proliferation of round-to-ovoid tumor cells with scant cytoplasm and a high nucleus-to-cytoplasm ratio. Nuclei was hyperchromatic with finely dispersed granular chromatin. Rosette structures and some ribbon-like patterns were seen (Fig. 2b). No necrosis was identified. There were three tumorlets around the tumor. Vascular and lymphatic invasion were present. The mitotic count was 13/2 mm^2^.

Immunohistochemically, the tumor cells were positive for chromogranin A, synaptophysin, CD56, and TTF-1 (Fig. 2c, d) and the Ki-67 index was 27% (Fig. 2e). The tumor cells were absent of immunoreactivity of SSTR-2 (score 0) (Fig. 2f). According to the current WHO classification, we diagnosed this tumor as SCLC with the pathological stage of IA2 (i.e. T1bN0M0).

She did not receive adjuvant chemotherapy. She was followed up by regular evaluations. As of 28 months after surgery, she remains in good health without relapse of lung cancer.

## Discussion

SCLC is likely to occur in males and has the strongest relationship with smoking [2]. SCLC usually shows a diffuse growth pattern, without an obvious neuroendocrine morphology, such as organoid, rosette, palisading, and trabecular patterns [2]. The WHO 2015 classification of lung tumors describes that a tumor with carcinoid morphology and mitotic counts > 10/2 mm^2^ is best classified as LCNEC [2], so the differential diagnosis of our cases was LCNEC. Differentiation between LCNEC and SCLC is sometimes difficult, reportedly due to morphological overlap between the two [5, 6]. In a routine diagnostic scene, we emphasize cytological findings, particularly nuclear findings, over tumor growth patterns such as a diffuse growth pattern or organoid pattern, when discriminating between SCLC and LCNEC. Our tumor cells had a high nucleus-to-cytoplasm ratio, low cytoplasm, and fine granular nuclear chromatin, all of which are seen in SCLC. Therefore, we diagnosed the two cases as SCLC. However, we consider that our tumors are not typical of SCLC due to the following four reasons: 1. The tumors occurred in female never-smokers. 2. Histology showed carcinoid morphology without extensive necrosis. 3. Proliferative abilities were extremely low compared to SCLC. 4. The tumors seemed to be biologically less aggressive than SCLC.

Quinn et al. reported twelve cases of lung NEC with carcinoid morphology with mitotic counts > 10/2 mm^2^ [7]. In their report, the average mitotic count was 25/2 mm^2^ and all tumors were completely resected. Eleven patients had recurrence and seven patients died from the tumor. For the seven metastatic cases, four patients were treated with platinum-based chemotherapy with no apparent response, whereas three other patients were treated using a combined therapy of capecitabine and temozolomide against well-differentiated gastroenteropancreatic NETs, of whom two showed good response. They reported that the clinicopathological features of these tumors seemed to be more akin to carcinoid than to LCNEC. Huang et al. classified AC into low- and high-grade AC [8]. Low-grade AC shows carcinoid morphology with the mitotic count ≤10/2 mm^2^ and high-grade AC shows carcinoid morphology with non-massive necrosis with the mitotic count > 10/2 mm^2^. They reported five-year overall survival rates of 83% for low-grade AC, 70% for high-grade AC, 60% for LCNEC and 40% for SCLC. According to their criteria, our cases may correspond to high-grade AC, a midgrade tumor.

In the WHO 2010 classification, gastroenteropancreatic NEN was divided into the following 3 grades based on mitotic count and Ki-67 index: NET grade 1 (NET G1) with mitotic count < 2/2 mm^2^ and/or Ki-67 index ≤2%, NET G2 with mitotic count 2 to 20/2 mm^2^ and/or Ki-67 index 3 to 20% and NEC with mitotic count > 20/2 mm^2^ and/or Ki-67 index > 20% [9]. Regarding pancreatic NEC, a new subtype of tumor was found that exhibits carcinoid-like morphology and shows clinicopathological features different from poorly differentiated tumor. Pancreatic NEC with carcinoid-like morphology has a high SSTR positive rate and is less responsive to platinum-based therapy but has better prognosis than poorly-differentiated tumor [10–12]. Based on these findings, pancreatic NEC was divided into carcinoid-like morphology type (NET G3) and poorly-differentiated type (NEC G3) in the WHO 2017 classification, and the diagnosis and treatment of pancreatic NEN was greatly changed [1]. Although the Ki-67 index of NET G3 is defined as exceeding 20%, it is usually ≤55%, and its proliferative activity is lower than that of NEC G3.

The WHO 2018 expert consensus proposed a new classification of NEN for other organs system including lung based on the same concept as the pancreatic NENs: 1. Well-differentiated tumors are classified as NET and poorly-differentiated tumors are classified as NEC based on the degree of differentiation. 2. According to proliferative ability, NETs are subclassified into G1, G2, and G3 [13]. In this proposed classification, TC and AC, well-differentiated tumors, correspond to NET G1 and NET G2, respectively, while SCLC and LCNEC, poorly-differentiated tumors, correspond to NEC [13].

Recently, regarding lung NETs, the WHO classification recommends the detection of immunohistochemical markers to confirm the neuroendocrine nature of tumor cells [2]. Commonly used markers include neuroendocrine markers (chromogranin A, synaptophysin, CD56). Most lung carcinoids and 80–90% of SCLCs are positive for neuroendocrine markers [2]. The tumors we studied were strongly positive for chromogranin A, synaptophysin, and CD56. Tumor expression of TTF-1 may also be utilized to aid in the diagnosis of lung NETs. TTF-1 is usually absent in lung carcinoids but positive in approximately 90% of SCLCs [2]. These immunohistochemical markers can be used when the histologic features are considered equivocal or the pathologist is looking for additional information. However, these markers cannot distinguish between subtypes of lung NETs. SSTR-2 exhibits mainly membranous immunostaining, and its expression is important for the diagnosis and management of patients with NEN [14]. SSTR-2 immunostaining was scored by Volante et al. as follows [4]: score 0: absence of immunoreactivity; score 1: pure cytoplasmic immunoreactivity, either focal or diffuse; score 2: membranous reactivity in less than 50% of tumor cells, irrespective of the presence of cytoplasmic staining; score 3: circumferential membranous reactivity in more than 50% of tumor cells, irrespective of the presence of cytoplasmic staining. According to this report, scores of 0 or 1 were considered negative, while scores > 1 were considered positive for SSTR-2 expression. It is reported that positive rate of SSRT-2 staining in pancreatic NET G3 is significantly higher than that in NEC G3, and its expression is considered to be indicative of good prognosis [10, 15, 16]. Tsuta et al. reported that in lung TC, AC, LCNEC and SCLC, immunoexpression of SSTR-2 was observed in 96.6, 77.8, 60 and 69% of cases, respectively. They suggested that SSTR-2 showed a tendency toward decreased expression in well- to poorly-differentiated tumors [17]. Case 1 was positive for SSTR-2 with score “2”, whereas case 2 was negative with score “0”. The expression of SSTR-2 in lung NEC with carcinoid morphology remains unclear. Ki-67, which may help separate lung carcinoid from LCNEC and SCLC, is only recommended as a complementary tool in the differential diagnosis of lung NETs. Although a grading system based on the Ki-67 index has previously been proposed [18, 19], it has not yet been included in the current WHO classification of the lung. Ki-67 index < 20% is in favor of carcinoid, while NECs show much higher proliferative activity than carcinoids, that is, Ki-67 index of SCLC is 50 to 100% (averaging ≥80%) and that of LCNEC is 40 to 80% [2]. The Ki-67 index of the tumors we studied were extremely low compared to SCLC.

SCLC has a poor prognosis with a median overall survival time of 12.9 months for limited disease [20]. This poor prognosis reflects the rapid growth of SCLC, its propensity for spreading to lymph nodes and distant organs, and the higher proportion of advanced disease presenting at diagnosis. Only 4% of patients present with a solitary nodule, and the rate of surgical resection is 1–6% [21, 22]. The cases we presented here were diagnosed at a resectable stage, and without adjuvant chemotherapy following surgery, the patients did not have a recurrence for almost two years, suggesting that these tumors may have less aggressive behavior than SCLC.

It is reported that lung NETs with carcinoid morphology having a mitotic count of > 10/2 mm^2^ corresponding to NET G3 are rare, not well characterized and need further study [7, 13]. The clinical and pathological features of our tumors seemed to be different from those of SCLC and they may fall into the category of NET G3. We consider identification of this subset to be relevant for therapeutic management.

## Conclusion

We presented two cases of lung neuroendocrine carcinoma with carcinoid morphology. There is no NET G3 category in the current WHO 2015 classification of lung tumors. However, we consider that our tumors may match the category of NET G3 and may be called “lung NET G3”. It is important to identify this subset for further therapeutic management.

## Data Availability

The data used and/or analysed during the current study are available from the corresponding author on reasonable request.

## Abbreviations

AC: Atypical carcinoid
CT: Computed tomography
FDG-PET: 18F-fluorodeoxyglucose-positron emission tomography
LCNEC: Large cell neuroendocrine carcinoma
NEC: Neuroendocrine carcinoma
NEN: Neuroendocrine neoplasm
NET: Neuroendocrine tumor
proGRP: pro-gastrin-releasing peptide
SCLC: Small cell lung carcinoma
SSTR: Somatostatin receptor
SUVmax: The maximum standardized uptake value
TBLB: Transbronchial lung biopsy
TC: Typical carcinoid
TTF-1: Thyroid transcription factor-1
WHO: World Health Organization
